# Determination of metal oxide and metallic nanoparticles in indoor air samples using mixed cellulose esters filters and spICP-MS: dissolve and shoot

**DOI:** 10.1007/s00604-025-07139-4

**Published:** 2025-04-08

**Authors:** Carlos Gómez-Pertusa, M. Carmen García-Poyo, Guillermo Grindlay, Ricardo Pedraza, Adela Yañez, Luis Gras

**Affiliations:** 1 Department of Analytical Chemistry, Nutrition and Food Sciences, University of Alicante, PO Box 99, 03080 Alicante, Spain; 2 Labaqua S.A.U., C/ Dracma, 16-18, Polígono Industrial Las Atalayas, 03114 Alicante, Spain

**Keywords:** Nanoparticles, Indoor air, Microwave extraction, Single particle analysis, Inductively coupled plasma mass spectrometry

## Abstract

In recent years, there has been increasing concern about the adverse health effects of the metallic and metal-containing nanoparticles (NPs) present in indoor environments. Unfortunately, there is no well-stablish method to simultaneously characterize their number and composition. Recently, our research group proposed a strategy for the determination of metallic nanoparticles in air by means spICP-MS based on the aerosol collection on micro-quartz filters and the subsequent extraction using microwave heating in basic media. Although the proposed method allows accurate and precise characterization of NPs, it suffers from practical drawbacks: (i) micro-quartz filter fibers are released into the sample and must be removed prior to analysis to avoid clogging the nebulizer and (ii) the particle distribution detection limits (LOD_size_) achieved are not low enough (28 nm). In this work, we evaluate the NPs trapping capabilities and possible fiber release of filters of different nature commonly used for indoor air quality control (polytetrafluoroethylene (PTFE), nylon, polycarbonate, and mixed cellulose ester (MCE) filters) and NPs of different chemical composition (ZrO_2_-, TiO_2_-, Pt-, AuNPs), size (20–150 nm), and capping agent (citrate, polyethylene glycol, branched polyethyleneimine, and lipoic acid). The results show that MCE is an optimal solution because it is completely dissolved during the microwave heating step and NPs are recovered quantitatively irrespective of their composition and size. The LODs are also improved down to 15 nm and 120 particles per liter of air, low enough to be used for indoor air pollution control. Finally, the proposed method was successfully tested in a simulated (NPs enriched) indoor environment.

**Graphical abstract:**

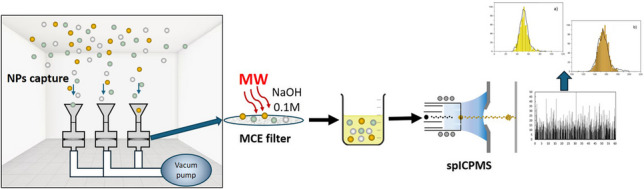

**Supplementary Information:**

The online version contains supplementary material available at 10.1007/s00604-025-07139-4.

## Introduction

The World Health Organization considers that air pollution is the contamination of the indoor or outdoor environment by any chemical, physical, or biological agent that modifies the natural characteristics of the atmosphere. Moreover, it is considered that air pollution can cause respiratory diseases and increases morbidity and mortality [1]. People in developed countries spend more than 80% of their time indoors, so most exposure to air pollution occurs in these environments. The particulate matter (PM) plays, together with other organic or inorganic phase gas pollutants, a significant role in indoor air quality. For this reason, there are well-established methods for the determination of particles with diameters of 10 and 2.5 microns (PM10 and PM2.5) [2]. However, in recent years, there has been increasing concern about the adverse health effects of the so-called ultrafine particles and engineered nanoparticles (NPs) [3] because their nanoscale dimensions make them capable of penetrating biological barriers and interacting with cells in potentially harmful ways. Both metallic and metal-containing NPs are widely used in applications ranging from catalysis to cosmetics [4, 5], and they are of particular concern due to their potential toxicity and ability to persist in indoor air both, in domestic or industrial workplaces [6–10]. For that reason, NPs effective monitoring and characterization is essential to assess their health and environmental impacts.

Filter-based sampling methods for indoor PM include both active techniques, where air is drawn through a filter and particulate matter is retained on the filter, and passive approaches, which rely on the natural deposition of the particles on the filter over time. Active methods, in particular, are widely used due to their higher precision and ability to quantify airborne particles in real-time. However, measuring NMs in such settings presents significant challenges due to their low concentrations and the complexity of their interactions with the environment. Current methodologies for NM characterization in air include direct methods, such as scanning mobility particle sizers (SMPS) or condensation particle counters (CPC), which provide real-time data on particle size distribution and concentration [11, 12]. However, these methods do not offer chemical composition insights and are significantly more expensive than filter-based ones. To gain chemical insights into airborne NM, some studies have recently explored methods that combine one of those techniques (SMPS or CPC) with inductively coupled plasma mass spectrometry in single particle mode (spICP-MS).

These hyphenations have proved effective not only in characterizing number concentration and size distribution but also in chemical analysis. However, their application to direct air samples is limited due to the sensitivity losses observed because of the presence of air in the argon, which affects sensitivity [13, 14]. To address these limitations, a method combining capture on micro-quartz filters, MAE in basic medium with subsequent spICP-MS analysis of non-labile metallic NPs has been recently developed by our research group [15, 16]. The filter-based methods are straightforward, easy to standardize, and robust, offering advantages for indoor air monitoring where consistency and reproducibility are critical. However, the use of micro-quartz filters for NP capture has revealed practical drawbacks due to the degradation of the quartz fibers during the microwave heating step. The released fibers block the nebulizer and also reduce the reliability of the method at low particle concentrations. In addition, this methodology has only been tested with citrate-stabilized metallic NPs (Au, Pt), and it is unclear whether it is also applicable to metallic NPs stabilized with other capping agents (e.g., amino and carboxylic), as well as other widely used NPs such as oxides metal oxides ones (Zr, Ti,…) [17, 18]. These limitations require the search for alternative filter materials that maintain robustness and reproducibility while supporting the development of standardized methods for indoor analysis of NPs of different nature (metallic and oxides). Among the alternative filters to quartz usually employed for the PM or the multi-elemental analysis of air, PTFE, Nylon, or polycarbonate are characterized by their chemical resistance that make them resistant to dissolution during the extraction step. On the contrary, MCE filters seem to be a promising alternative due to their complete solubilization during sample preparation step [19, 20]. In any case, the release of fibers should be eliminated and the sample throughput improved.

The aim of this work was to develop a robust and reproducible methodology for NP analysis in indoor environments using spICP-MS. To this end, a MAE protocol was applied to extract the NPs from filters of different materials and NPs of different nature (metallic, oxides) and characteristics (size and capping agent). Subsequently, the capture efficiency of the filters was evaluated, and the methodology was tested in a simulated (NPs enriched) indoor environment to demonstrate its applicability under standard capture conditions for air quality monitoring.

## Materials and methods

### Reagents

All solutions used in this study were prepared with ultrapure water from a Milli-Q purification system (Millipore Inc., France). The stock suspensions of metallic and oxide metallic NPs of different characteristics (i.e., size and caping agent) utilized throughout this work are listed in Table 1.

**Table 1:** NP characteristics and suppliers

NP nature	Size/nm	Concentration/NPs mL^−1^	Capping agent	Supplier
Pt	70	1.2 × 10^10^	Citrate (CIT)	NanoComposix (San Diego, USA)
Au	20	2 × 10^11^	Citrate (CIT)	NanoComposix (San Diego, USA)
	50	3.5 × 10^10^	Citrate (CIT)	Cytodiagnostics (Burlington, Canada)
	100	3.9 × 10^9^	Citrate (CIT)	Cytodiagnostics (Burlington, Canada)
		1.0 × 10^11^	Polyethylene glycol (PEG)	NanoComposix (San Diego, USA)
		1.3 × 10^11^	Branched polyethyleneimine (BPEI)	NanoComposix (San Diego, USA)
		9.2 × 10^10^	Lipoid acid (LIP)	NanoComposix (San Diego, USA)
	150	3.6 × 10^9^	Citrate (CIT)	Cytodiagnostics (Burlington, Canada)
ZrO_2_	30	5.9 × 10^12^	-	Sigma-Aldrich (Steinheim, Germany)
TiO_2_	150	4.2 × 10^10^	-	Glantreo (Cork, Ireland)

For calibration purposes in spICP-MS size measurements, platinum, gold, zirconium, and titanium mono-elemental standards (1000 mg L^−1^) from Sigma-Aldrich were used to create calibration curves with known mass concentrations. And 99.996% w w^−1^ sodium hydroxide from Thermo-Fisher Scientific (Waltham, USA) was also used for preparing the extraction medium.

### Transmission electron microscopy (TEM)

Transmission electron microscopy (TEM) was used to characterize the NP size distribution and also to validate the accuracy of spICP-MS data. Methodology has been previously described [15]. Briefly, the nanoparticle suspension was sonicated for 30 s, and a single drop was deposited onto TEM copper/carbon grids. The sample was air-dried for 10 min before direct observation using a 120 kV JEM-1400Plus electron microscope (JEOL, Tokyo, Japan).

### Filters

Five filters of different material and physical characteristics were used in this work: (i) Micro-quartz-fiber (50 mm diameter, 0.3 mm nominal pore size, Thermo-Fisher Scientific (Waltham, USA)), (ii) Nylon (37 mm, 0.8 mm nominal pore size, Thermo-Fisher Scientific (Waltham, USA)), (iii) PTFE (37 mm, 0.8 mm nominal pore size, Thermo-Fisher Scientific (Waltham, USA)), (iv) Polycarbonate (47 mm, 0.22 $\mu m$ nominal pore size, Sigma Aldrich (Barcelona, Spain)), and (v) MCE (37 mm, 0.8 mm nominal pore size, SKC (Pittsburgh, USA)).

### Evaluation of NPs capture by the filters

To evaluate the efficiency of NPs capture by the filters, a suspension with a known concentration of 10^7^ NPs mL^−1^ was nebulized for 10 min under controlled conditions using a conventional ICP sample introduction system made up by a concentric nebulizer and a 40 mL cyclonic spray chamber. The sample uptake rate (*Q*_1_) was set to 100 μL min^−1^, and the nebulizer gas flow rate (*Q*_*g*_) was fixed at 1.0 L min^−1^ to maximize the aerosol transport. Aerosols were collected at the spray chamber’s exit using a vacuum pump model GM-0.50 from Comevta-Yvimen (Barcelona, Spain) working at 9 L/min of capture flow. This methodology has been employed as an indoor capture proxy because (i) it has been previously tested by Torregrosa et al. [15] using wet and dry aerosols and (ii) more than 92% of the droplets at the exit of the spray chamber are below 1.2 $\mu m$ (similar magnitude than NPs) and, therefore, the NPs capture filter efficiency should not be significantly different from that obtained for liquid droplets, whether or not they are contained in droplets. Afterward, the NPs were extracted from the filters using a suitable extraction method, and the supernatant was analyzed by ICP-MS. To ensure that NPs were retained in the filter, a second filter was added to the setup as a control [15].

### Microwave-assisted extraction methodology

Microwave-assisted extraction (MAE) of NPs from filters was performed in the same way as described previously for micro-quartz air filters [15, 16]. NPs were spiked on the filters and dried overnight; then, MAE was performed using the previously developed MW program with the MW digester (Milestone, Ultraclave). Once the program was complete, and samples reached room temperature were diluted to 40 mL of final volume, centrifugated (when necessary) to separate the NPs from the filter fibers, and finally, the supernatant was ultimately analyzed by spICP-MS. Platinum NPs were selected for this study due to their exceptional stability, minimal memory effects, negligible spectral interferences, and the generation of a low background signal [21].

### ICP-MS instrumentation

A triple-quadrupole based 8900 ICP-MS from Agilent Technologies (Santa Clara, USA) was used throughout this work. Table 2 summarizes operating conditions. In the case of TiO_2_NPs, particular attention must be paid to the potential for isobaric interferences, primarily those arising from ^48^Ca, and matrix-based polyatomic interferences, including those derived from C, S, and P [22]. To address these interferences, the use of O_2_ (0.3 mL min^−1^) was used as the reaction gas in the collision/reaction cell. The first quadrupole was set to m/z 48 (^48^Ti), while the second quadrupole was set to m/z 64 (^48^Ti^16^O). Additionally, H_2_ (9.0 mL min^−1^) was employed to generate the ion ^48^Ca^16^O^1^H, thereby mitigating the interference of the ^48^Ti^16^O ion, formed by the O_2_ gas with the ^48^Ca^16^O.

**Table 2:** ICP-MS operating conditions

	Single particle mode
Plasma forward power (W)	1550
Sampling depth (mm)	8^#^; 10^$&^
Argon flow rate (L min^−1^)	
Plasma	15
Auxiliary	0.9
Nebulizer (Q_g_)	1.00
Oxygen flow rate (mL min^−1^)	0.3^$^
Hydrogen flow rate (mL min^−1^)	9.0^$^
Torch i.d. (mm)	1.0
Sample introduction system	
Nebulizer	MicroMist® nebulizer
Spray chamber	Scott double pass
Sample uptake rate (*Q*_*l*_) (μL min^−1^)	300
Dwell time (ms)	0.1
Measuring time (s)	60
Nuclides	^48^Ti;^90^Zr;^195^Pt;^197^Au

^$^Ti; ^&^Zr; ^#^Pt and Au

Data acquisition and analysis were performed using the single nanoparticle application module in the ICP-MS control software (MassHunter version 4.5). Calibration for single particles was achieved through the frequency method described by Pace et al. [23]. This approach enables precise determination of transport efficiency by counting the number of events recorded from a nanoparticle suspension with a known concentration, provided the plasma remains stable during operation [21].

### Indoor experiment

To evaluate the applicability of the developed methodology, a closed room experiment under controlled conditions was designed (Fig S1; Supplementary). To this end, a NP suspension of known concentration (10^8^ NPs mL^−1^) was nebulized using a domestic ultrasonic nebulizer (Humiplus, Miniland Baby, Onil, Spain) at a nebulization flow rate (*Q*_neb_) of 7.5 mL min^−1^ during 10 min (*t*_neb_) in a closed room with a volume of 52 m^3^ at a fixed temperature of 25 °C and a relative humidity of 75%. After this time, different (i) capture time (30 and 60 min), (ii) type of particles (nature and size), (iii) capture flow rate (2.7 and 8.2 L/min), and (iv) NP concentration (1·10^7^–5·10^8^ NPs mL^−1^) were studied to check if the methodology is able to detect the changes produced in the closed room system.

## Results and discussion

### NP extraction from the filters

The first step was to investigate the effect of filter nature on nanoparticle extraction and detection. To this end, a known quantity of PtNPs (0.2 g of 4·10^6^ mL^−1^) was spiked on the different filters studied and then extracted using the method developed in a previous study with 10 mL of NaOH 0.1 M as extractant for 10 min at 1200 W [16]. Table 3 shows the particle number recovery obtained for the different filters and NPs after the treatment. The results in Table 3 confirm the quantitative recoveries of PtNPs observed in previous studies when using the micro-quartz filters [15, 16]. Regarding the other filters tested, no quantitative recoveries were obtained when using nylon, polycarbonate, or PTFE. Changes in extraction conditions (time and/or NaOH concentration) or a second extraction do not significantly improve NP recovery. In the case of nylon and polycarbonate, this is probably because the filters melt during the microwave extraction step, preventing the release of the NPs. A second extraction also fails to extract the retained NPs from the melted filter. On the contrary, the MCE filters provide quantitative recoveries under the extraction conditions tested. These filters represent a notable alternative to micro-quartz due to their widespread use in environmental air quality monitoring, particularly for the collection of particulate matter, and meet the requirements of current regulations [24–26]. Moreover, it is of particular significance to note that MCE filters undergo dissolution during the microwave heating process, a phenomenon that offers additional advantages. The complete filter dissolution avoids the centrifugation step and the nebulizer blockage (In fact, the nebulizer remained unclogged throughout the course of this study) simplifying the method and increasing the sample throughput. In addition, the complete dissolution of the filter also prevents the possible retention of NPs within the filter, increasing the robustness of the method. For all these reasons, MCE was selected as the most promising material for recovering NPs from filters, but further studies are needed to evaluate its ability to capture and recover NPs of different characteristics (i.e., nature, size, and capping agent) in indoor environments.

**Table 3:** Particle number recovery of NPs in different filter materials after microwave treatment. Data expressed as the mean value ± s (standard deviation), *n* = 5

NP nature	Nominal size/nm	Capping agent	Filter	Recovery/%	spICP-MS size/nm	TEM size/nm
Pt	70	CIT	Micro-quartz	97 ± 6	68.5 ± 0.8	69 ± 2
			Nylon	13 ± 2		
			Polycarbonate	45 ± 3		
			PTFE	72 ± 5		
			MCE	96 ± 5		
Au	100	CIT	MCE	97 ± 5	94.1 ± 0.5	102 ± 8
ZrO_2_	30	-		103 ± 4	28 ± 3	27 ± 8
TiO_2_	150	-		96 ± 6	125 ± 15	140 ± 40
Au	20	CIT	MCE	96 ± 6	22 ± 2	19 ± 2
	50			99 ± 3	48.5 ± 0.5	52 ± 6
	150			96 ± 6	145.3 ± 0.7	155 ± 12
	100	PEG	MCE	95 ± 5	98.8 ± 0.5	100 ± 6
		BPEI		96 ± 6	98.1 ± 0.6	98 ± 4
		LIP		97 ± 4	97.4 ± 1.1	102 ± 8

Capping agent: *CIT*, citrate; *PEG*, polyethylene glycol; *BPEI*, branched polyethyleneimine; *LIP*, lipoid acid

To this end, NPs of different nature: gold and metal oxide NPs stable under the basic extraction media employed (ZrO_2_-TiO_2_NPs) were first evaluated. Results obtained, as observed previously with PtNPs, show quantitative recoveries in all the cases (Table 3). In the same way, AuNPs of different sizes (i.e., 20, 50, 100, and 150 nm) and capping agents (i.e., CIT, PEG, BPEI, and LIP) were investigated and quantitative results were also obtained in all cases (Table 3). It can therefore be concluded that, under the microwave heating conditions employed, the use of MCE filters allows the quantitative recovery of NPs regardless of their nature (metal or metal oxide), size, or capping agent.

**Fig. 1:**
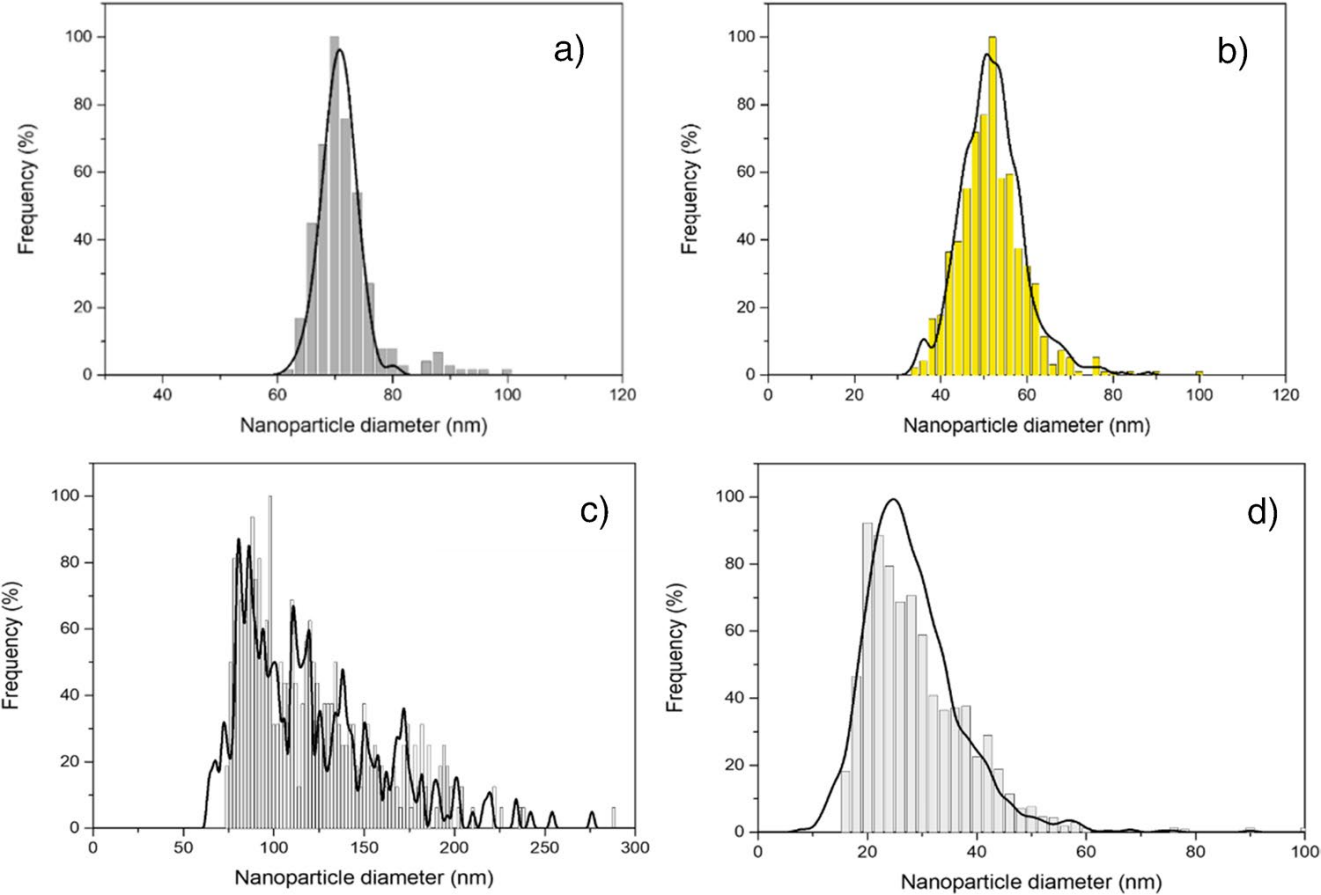
Nanoparticle size distributions by means TEM (black line) and after microwave assisted extraction (colored bars) obtained by spICP-MS for: **a** 70 nm PtNPs, **b** 100 nm AuNPs, **c** 150 nm TiO_2_NPs, and **d** 28 nm ZrO_2_NPs

Once the quantitative recovery of NPs has been confirmed, it is also necessary to verify that the size of the NPs is not modified during the microwave heating step. To this end, a comparison was made between the particle size distributions obtained after extraction and those obtained by TEM before treatment. Figure 1 shows the size distributions obtained by both techniques. In general, the size distribution of the particles remains unchanged after extraction, regardless of their size and nature. However, it should be noted that in the case of oxides (Fig. 1c and d), small differences can be observed between the techniques at the smallest diameters. These differences are related to the lack of sensitivity for these oxides obtained with the triple quadrupole ICPMS used and not to the filter treatment itself. The use of more sensitive spectrometers such as sector field ICPMS could avoid this difference [27]. Similar results were obtained when evaluating capping agents and size (Figures S2-S3).

### NPs capture efficiency by the MCE filters

Following the confirmation of the quantitative recovery of NPs from the MCE filters and the verification of their integrity, the subsequent study focused on the capture of NPs within the filter. To this end, following the work of Torregrosa et al., a 10^7^ NPs mL^−1^ of NPs of different characteristics (i.e., nature, size, and capping agent) were nebulized, and the aerosol emerging of the spray chamber was captured using two MCE filters (Fig. S4, supplementary) [15]. In order to evaluate if the NPs pass through the filter or if they are retained, a second filter was placed into the system. The number of NPs captured in the filters was calculated considering the transport efficiency of the ICP sample introduction system employed [15].

Results shown in Table 4 indicate that all the NPs, irrespective of their characteristics (nature, size, and capping agent), are quantitatively retained in the first filter, being the quantity of the NPs retained in the second filter as low as the experimental error. These results can be explained attending to the high electrostatic charge of the MCE filters and confirms, again, their suitability for NPs analysis purposes in air.

**Table 4:** Percentage of the NPs retained in the first and the second MCE filters. Results are expressed as the mean value ± s (standard deviation), *n* = 5

NP nature	Nominal size/nm	Capping agent	% NPs_Filter 1_	% NPs_Filter 2_
Pt	70	Citrate	99 ± 2	1.2 ± 0.2
Au	100	Citrate	100 ± 3	0.18 ± 0.06
ZrO_2_	28	-	99 ± 4	0.90 ± 0.09
TiO_2_	140	-	100 ± 5	0.13 ± 0.02
Au	20	Citrate	101 ± 3	0.08 ± 0.02
	50		98 ± 2	0.09 ± 0.04
	100		100 ± 3	0.18 ± 0.06
	150		97 ± 5	0.20 ± 0.15
	100	Polyethylene glycol	98 ± 4	0.13 ± 0.02
		Branched polyethyleneimine	99 ± 4	0.19 ± 0.02
		Lipoid acid	100 ± 3	0.12 ± 0.05

### Limits of detection (LODs)

**Fig. 2:**
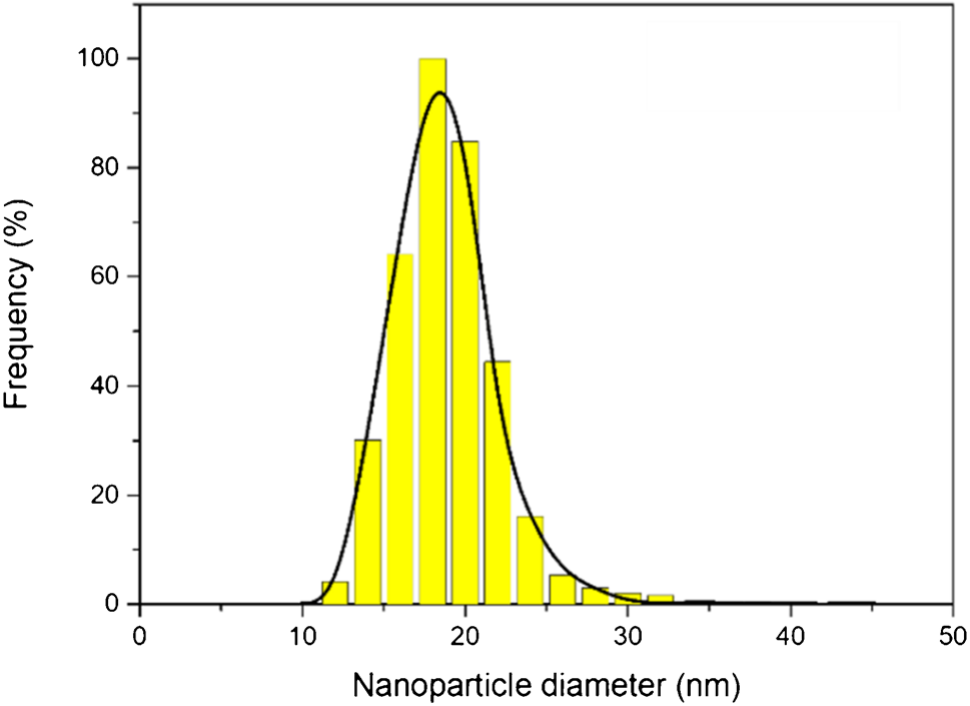
20 nm AuNP size distribution by means TEM (black line) and after the capture in MCE filters and MAE obtained by spICP-MS (bars)

The complete dissolution of MCE filters following the microwave heating step may have a detrimental effect on the signal background. This, in turn, may have a negative effect on the size and particle number detection limits (LOD_size_ and LOD_part_). To address this, the signal background levels, LOD_size_ and LOD_part_, calculated based on the method proposed by Bolea et al. [28], obtained with MCE filters for NPs of different nature were compared to those from micro-quartz filters. The results shown in Table 5 demonstrate that the backgrounds and both LODs (size and number) achieved with MCE filters were comparable to those obtained with micro-quartz filters, regardless of the NP nature. The LOD_size_ obtained for Pt, Au, and ZrO_2_ are similar (17 nm on average), whereas the TiO_2_ is around three times higher. These values are consistent with those previously reported in the literature for quadrupole-based ICP-MS instruments and are attributed to the differences in instrument sensitivity and background levels among elements [28, 29]. As regards LOD_part_ (NPs L^−1^_air_, 81 L sampled), the values obtained for the metallic NPs are significantly lower than the obtained for oxides (up to 50 times) but, in all cases, are low enough to be used in real cases of indoor air pollution studies. To further verify the performance of the method, the particle size distribution of the smallest NPs studied (20 nm Au) was determined. The distribution obtained after capture and extraction using MCE filters and determination by spICP-MS was compared to the TEM reference (Fig. 2). The findings revealed no significant changes in particle size distribution, thus confirming the applicability of the LODs obtained. In cases where it is necessary to determine particles with sizes smaller than the LOD_size_ obtained with quadrupole-based instruments, sector field spectrometers, as it has been previously mentioned, could be employed [27].

**Table 5:** Background levels and LODs using micro-quartz and MCE filters for NPs of different nature after the microwave extraction. Results are expressed as the mean value ± s (standard deviation), *n* = 3

NP nature	Micro-quartz filter			MCE filter		
	Background/cps	LOD_size_/nm	LoD_part_/ NPs L^−1^_air_	Background/cps	LOD_size_/nm	LOD_part_/NPs L^−1^_air_
Pt*	3800 ± 300	16	120	4200 ± 300	16	120
Au*	4500 ± 400	15	130	4600 ± 400	15	130
ZrO_2_	8200 ± 700	19	600	9000 ± 600	19	500
TiO_2_	41,000 ± 4000	60	6000	45,000 ± 5000	60	6000

LoD_part_ obtained using 81 L air volume sampled. *Capping agent: citrate

### Indoor experiment

To evaluate the methodology under a simulated case scenario, a known concentration of NPs was nebulized into a controlled environment. Using an ultrasonic nebulizer, the particles were dispersed under the conditions described in the experimental Section "ICP-MS instrumentation" in a closed room maintained under controlled temperature and humidity. Following, the NPs were captured on MCE filters using a vacuum pump under different experimental conditions: (i) capture time, (ii) capture flow rate, (iii) NP nature, and (iv) NP concentration.

First, the effect of capture time on NP quantification was examined to determine the ability of the methodology to detect temporal variations in NP concentration. For this experiment, capture flow rate was fixed at 2.7 L min^−1^ and NP concentration at 5·10^8^ NPs mL^−1^. Two capture times were tested: 30 min, ensuring sufficient sampling, and 60 min, allowing additional time for environmental changes in the closed system.

The results showed that the number of NPs captured during 30 min (54 ± 6·10^3^ NPs_cap_) was comparable to that for 60 min (42 ± 12·10^3^ NPs_cap_).

Aerosol dynamics are rather complex, but our results suggest that the NPs are captured at the beginning of the experiment and, hence, an increase in the capture time does not affords higher NPs capture but increases uncertainty. It can therefore be concluded that, under our experimental conditions, the different aerosol phenomena shown by NPs (i.e., coagulation, sedimentation, and agglomeration [30–33]) take place for the entire duration of the aerosol capture time. As there is no additional NP input during the experiment (ultrasonic nebulizer is off), the longer the capture time, the greater the effect and the number of NPs captured is not increased.

Attending to these results, 30 min was selected as the capture time for subsequent experiments due to its better reliability.

In order to evaluate the relationship between the sampling flow rate and the efficiency of NPs of different nature and size capture (i.e., 20 nm AuNPs, 70 nm PtNPs, and 28 nm ZrO_2_NPs), experiments were conducted at two flow rates: 2.7 and 8.2 L min^−1^. The latter represented the maximum flow achievable with the calibrated vacuum system employed, while the former aligned with regulatory recommendations, respiration simulation, and multi-sample compatibility [24–26]. The results shown in Table 6 indicated a proportional relationship between the flow rate and the number of NPs captured. Specifically, tripling the flow rate led to a threefold increase in the number of NPs captured, regardless of the characteristics (nature and size) of the NPs nebulized. This confirmed a robust linear correlation between sampled air volume and detected NPs across different particle types. It is also important to remember that in this experiment, all the NPs (Au, ZrO_2_, and Pt) were nebulized simultaneously, which also highlights the ability of the methodology to detect different NPs in the same filter sample. This is an aspect to be considered when conducting studies in environments where the composition of the NPs to be sampled is not well known.

**Table 6:** NPs captured using two different capture flow rates. Results are expressed as the mean value ± s (standard deviation), *n* = 3

NP nature	Capture flow rate L min^−1^	
	2.7	8.2
Au* (20nm)	1.06 ± 0.10 × 10^6^	3.1 ± 0.2 × 10^6^
ZrO_2_ (28nm)	5.7 ± 0.2 × 10^5^	1.68 ± 0.08 × 10^6^
Pt* (70nm)	5.9 ± 0.4 × 10^4^	1.90 ± 0.06 × 10^5^

*Capping agent: citrate

Finally, the correlation between NPs nebulized and captured was evaluated. To this end, PtNPs at concentrations ranging from 1·10^7^ to 5·10^8^ NPs mL^−1^ were sprayed and the total number of NPs nebulized and captured are shown in Table 7. As it can be observed, the results show that the higher the NPs nebulized the higher the NPs captured and also that the capture efficiency decreases when the number of NPs nebulized does. Thus, for instance, an increase of 45 times in the number of NPs nebulized leads to 7.5 times increase in the number of NPs captured. These data, together with those previously observed when time and suction flow rate were varied, seem to indicate that the observed behavior is related to the aerosol dynamics and not to the sampling methodology.

**Table 7:** PtNPs captured for the different concentrations nebulized. Results are expressed as the mean value ± s (standard deviation), *n* = 3

NPs nebulized mL^−1^/10^7^	NPs captured mL^−1^/10^3^
1.10 ± 0.06	7.1 ± 0.3
4.8 ± 0.2	11.8 ± 0.4
11.3 ± 0.5	19.7 ± 0.3
45.4 ± 0.7	53.6 ± 0.5

## Conclusions

This work presents a straightforward methodology for the characterization of NPs in indoor environments using MCE filters in conjunction with spICP-MS. This approach eliminates the need for complex separation steps as the MCE filters completely dissolve during the microwave heating step, simplifying the process while increasing both robustness, sample throughput, and standardization. The method is effective for the detection of nanoparticle distributions larger than 15 nm (medium diameter), regardless of their material type (metal or metal oxide) or capping agent, making it suitable for a wide range of NPs. This versatility, combined with the improved ease of use, positions the method as a valuable tool for environmental monitoring and indoor air quality assessment, providing consistent and reliable results for different types of NPs. The method has been evaluated under simulated scenario (NPs enriched indoor environment) showing promising results, but future works under real environments should be done.

## Supplementary Information

Below is the link to the electronic supplementary material.

Supplementary file1 (DOCX 769 KB)

## Data Availability

No datasets were generated or analysed during the current study.
