# Supplementary material for: Determination of metal oxide and metallic nanoparticles in indoor air samples using mixed cellulose esters filters and spICP-MS: dissolve and shoot
Source: Mikrochim Acta. 2025 Apr 8;192(5):288. doi: 10.1007/s00604-025-07139-4 (PMC11978717; doi:10.1007/s00604-025-07139-4)
Supplement: Supplementary file 1 — Supplementary file1 (DOCX 769 KB) [file 604_2025_7139_MOESM1_ESM.docx]

**Supplementary material**

**
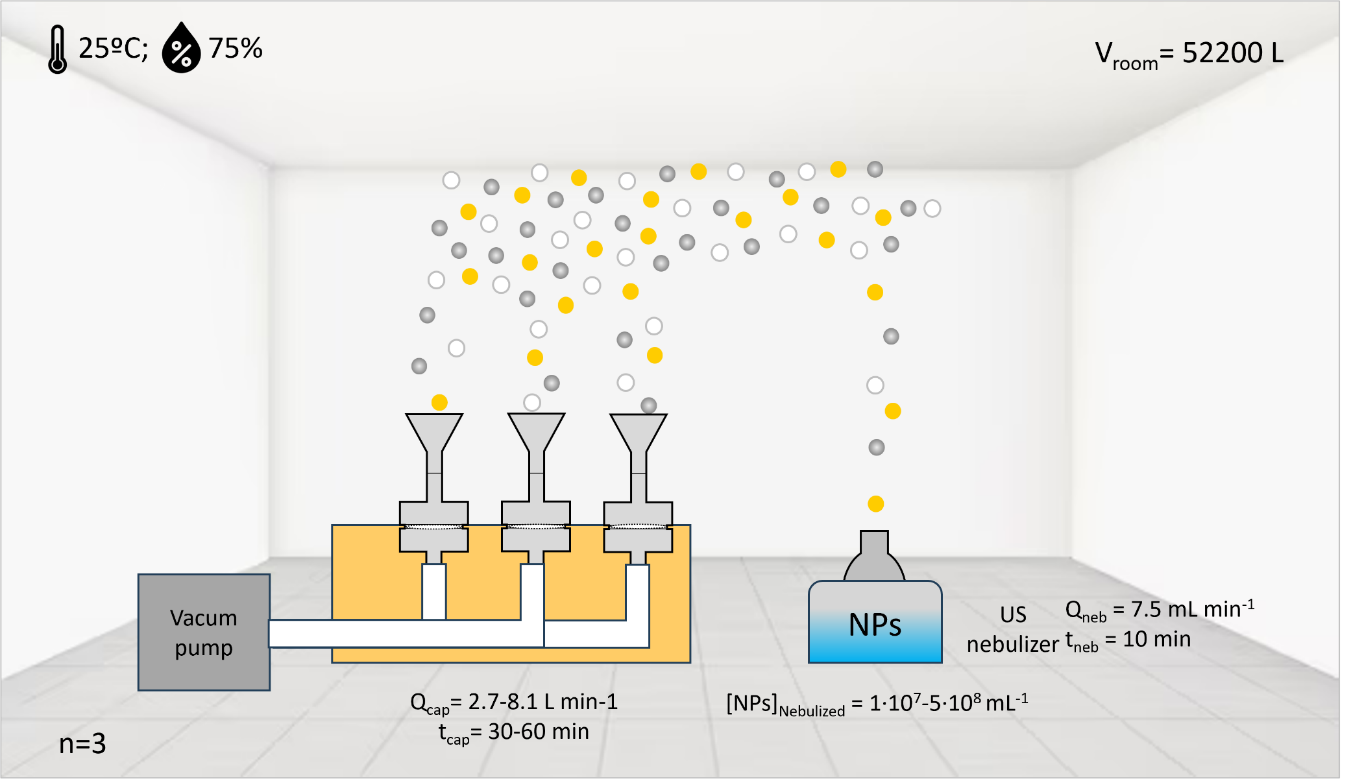
**

**Figure S1.** Experimental set up for the closed room experiment


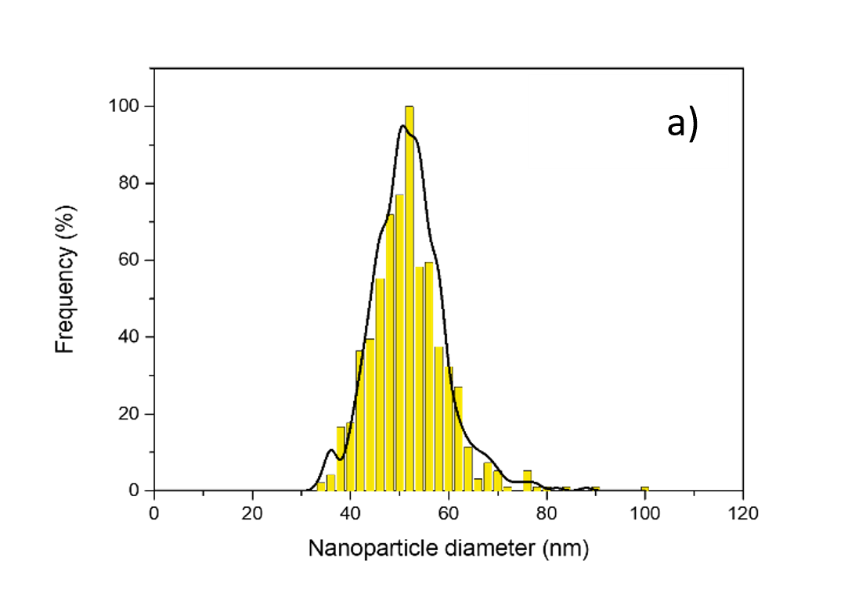
**
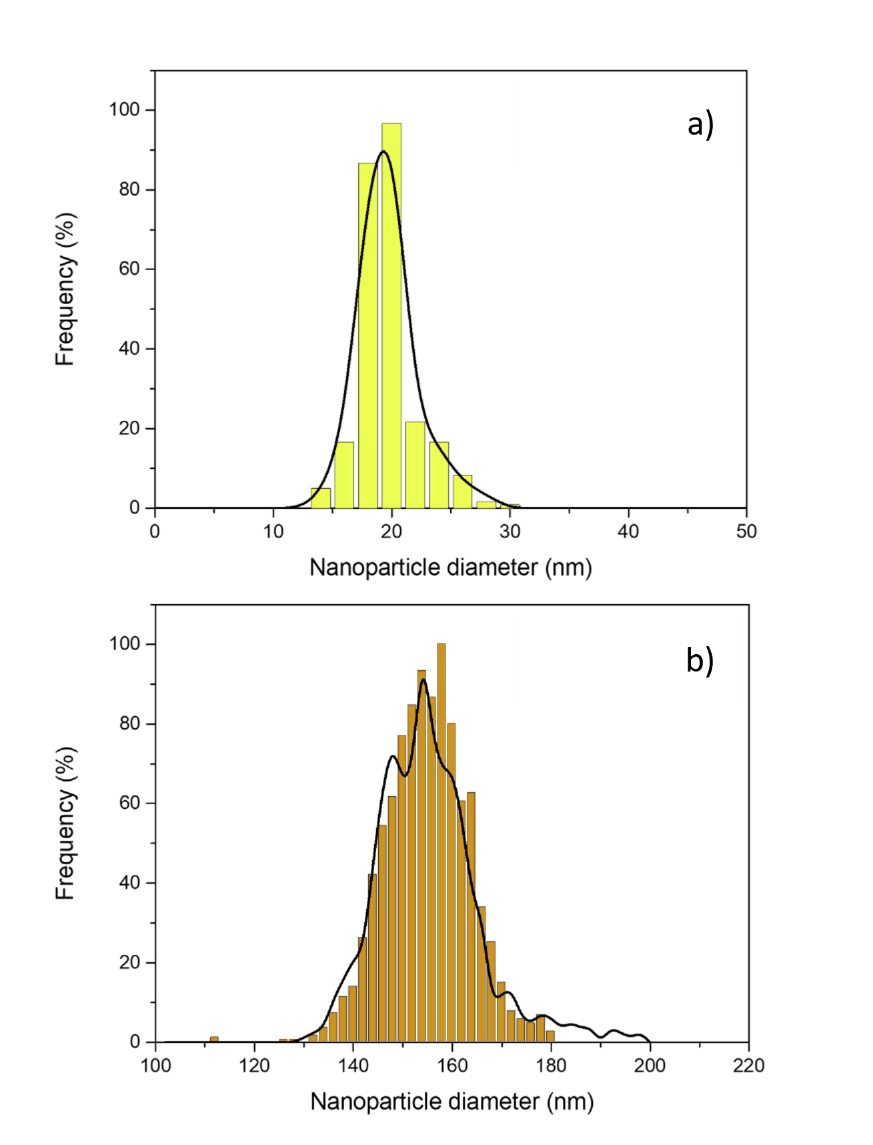
**

**Figure S2.** Nanoparticle size distributions for 50 (a) and 150nm (b) AuNPs. The continuous black line represents TEM particle size distribution and bars represent particle size distribution after MAE.


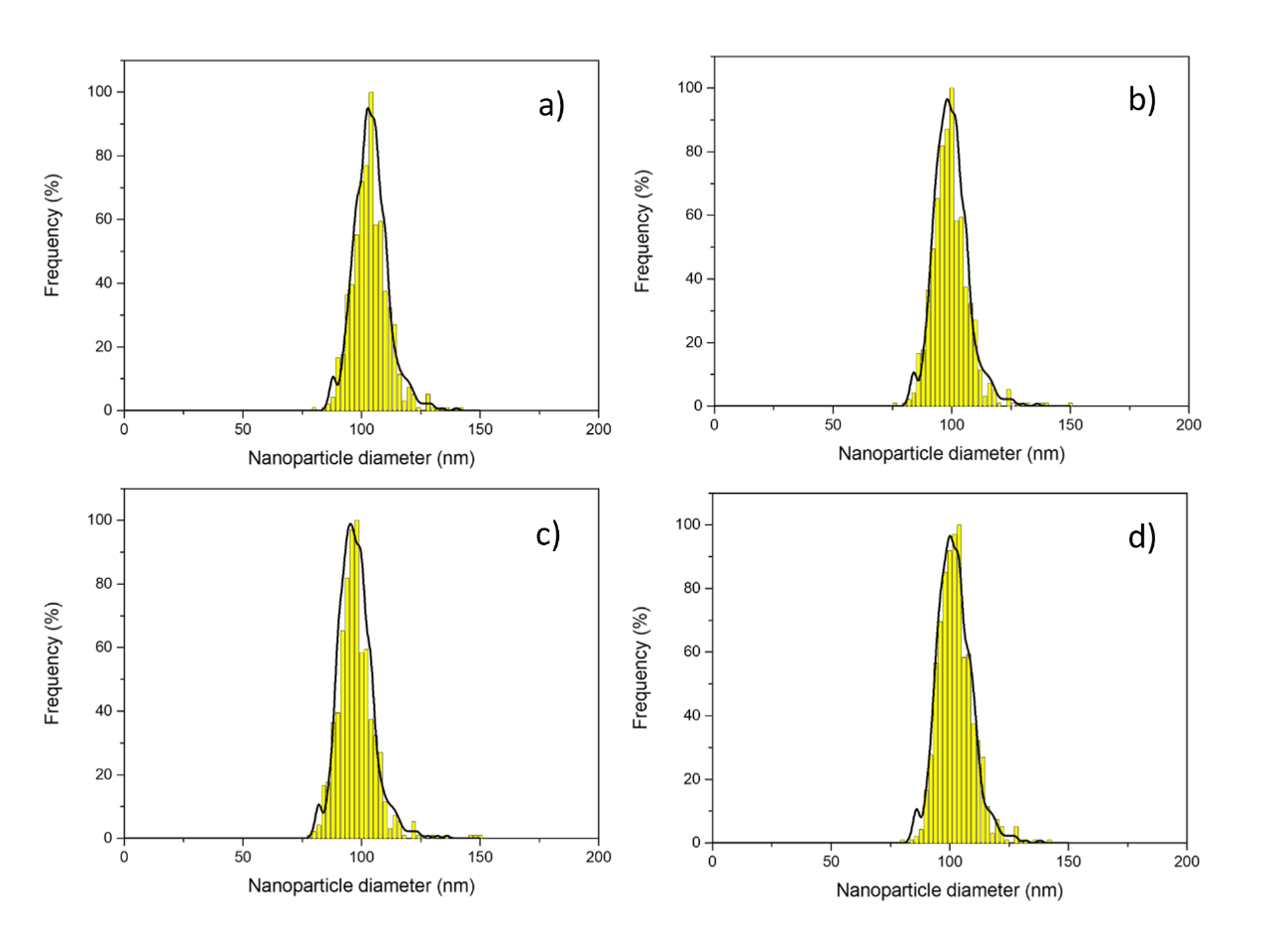


**Figure S3.** Nanoparticle size distributions for AuNPs of (a) CIT, (b) PEG, (c) BPEI and (d) LIP. The continuous black line represents TEM particle size distribution and bars represent particle size distribution after MAE.


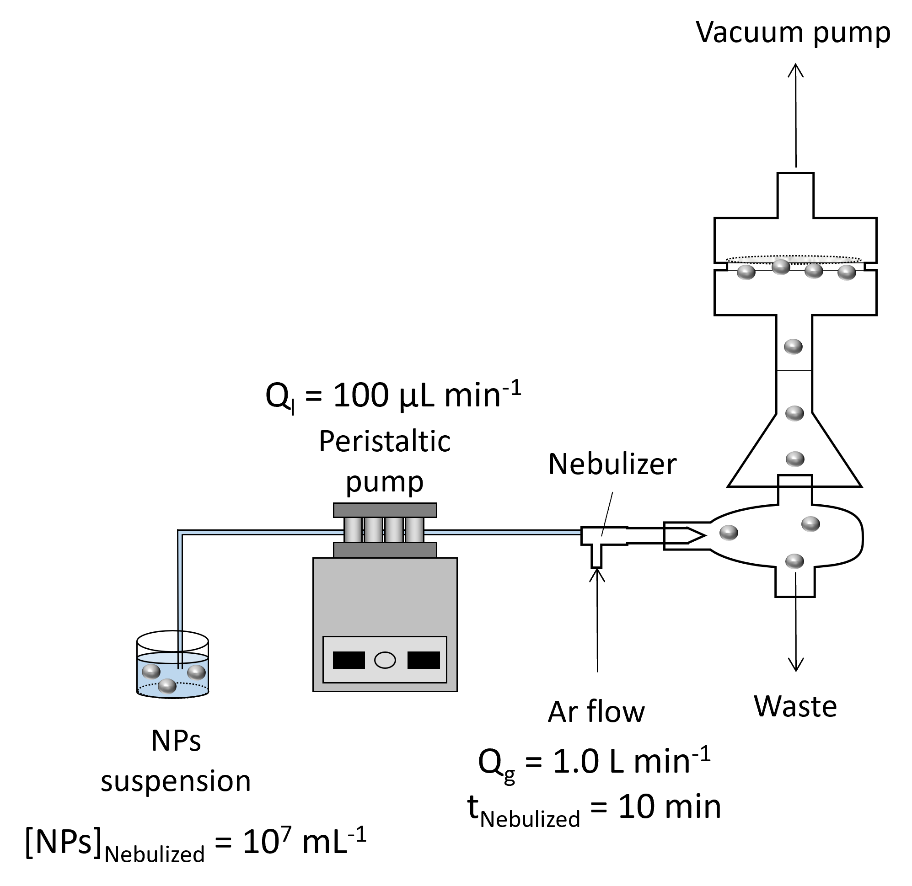


**Figure S4.** Experimental setup for studying NPs capture efficiency in the MCE filters.
